# Contemporary outcomes of left thoraco-abdominal esophagectomy due to cancer in the esophagus or gastroesophageal junction, a multicenter cohort study

**DOI:** 10.1093/dote/doae039

**Published:** 2024-04-28

**Authors:** F Klevebro, S Ash, C Mueller, G M Garbarino, S S Gisbertz, M I van Berge Henegouwen, Y Mandeville, L Ferri, A Davies, N Maynard, D E Low

**Affiliations:** Department for Thorqacic Surgery, Virginia Mason Medical Center, Seattle, WA, USA; CLINTEC, Karolinska Institute, Stockholm, Sweden; Oxford University Hospitals NHS, Ludwig Institute for Cancer Research, Nuffield Department of Medicine, University of Oxford Trust, Oxford, UK; Mc Gill University Health Center, Montreal, Canada; Department of Surgery, Cancer Center Amsterdam, Cancer Treatment and Quality of Life, Amsterdam UMC Location University of Amsterdam, Amsterdam, The Netherlands; Department of Medical Surgical Science and Translational Medicine, Sapienza University of Rome, Sant’ Andrea Hospital, Rome, Italy; Department of Surgery, Cancer Center Amsterdam, Cancer Treatment and Quality of Life, Amsterdam UMC Location University of Amsterdam, Amsterdam, The Netherlands; Department of Surgery, Cancer Center Amsterdam, Cancer Treatment and Quality of Life, Amsterdam UMC Location University of Amsterdam, Amsterdam, The Netherlands; AZ Delta, Roeselare, Belgium; Mc Gill University Health Center, Montreal, Canada; St Thomas’, King’s College London, London, UK; Oxford University Hospitals NHS, Ludwig Institute for Cancer Research, Nuffield Department of Medicine, University of Oxford Trust, Oxford, UK; Department for Thorqacic Surgery, Virginia Mason Medical Center, Seattle, WA, USA

**Keywords:** esophageal cancer, gastroesophageal junction cancer, left thoracoabdominal esophagectomy, postoperative complications

## Abstract

Surgery for cancer of the esophagus or gastro-esophageal junction can be performed with a variety of minimally invasive and open approaches. The left thoracoabdominal esophagectomy (LTE) is an open technique that gives an opportunity to operate in the chest and abdomen with excellent exposure of the gastro-esophageal junction through a single incision, and there is currently no equivalent minimally invasive technique available. The aim of this multi-institutional review was to study a large contemporary international study cohort of patients treated with LTE. An international multicenter cohort study was performed including all patients treated with LTE at six high-volume centers for gastro-esophageal cancer surgery between 2012 and 2022. Patient data were prospectively collected in each participating centers’ institutional database. Information about patient, tumor, and treatment details were collected. The study cohort included a total of 793 patients treated with LTE during the study period. The most frequently observed complications were pneumonia in 185/727 (25.5%) patients and atrial fibrillation in 91/727 (12.5%). Anastomotic leak occurred in 35/727 (4.8%) patients; no patient suffered from conduit necrosis. Thirty-day mortality occurred in 15/785 (1.9%) patients and 90-day mortality in 39/785 (5.0%) patients. Factors with statistically significant association with survival were American Society for Anesthesiologists-score, tumor location, tumor stage, and tumor free resection margins. Neoadjuvant therapy was not associated with increased survival compared to surgery alone but neoadjuvant chemoradiotherapy compared to neoadjuvant chemotherapy showed statistically significant improved survival with hazard ratio 0.60 (95% confidence intervals:0.44–0.80, *P* = 0.001) in a multivariable adjusted model. This study demonstrates that LTE can be applied in selected patients with results that are comparable to other large studies of open and minimally invasive surgery for esophageal or gastro-esophageal cancer at high-volume centers.

## INTRODUCTION

Surgical treatment for cancer of the esophagus and gastroesophageal junction usually requires a combined thoracoabdominal approach. The most important factors for successful treatment are to achieve tumor free resection margins, perform adequate lymph node resection, and to create a functional reconstruction that heals without leak.^1^^,^^2^ An esophagectomy for cancer can be performed with open or minimally invasive approach and is known to be associated with significant risk for postoperative complications and long-term reduced health-related quality of life for patients. Minimally invasive technique has in recent years become standard treatment at many high-volume centers in the Western world; however open esophagectomy is still required in selected patients due to large or bulky tumors or to create access for resection of non-regional lymph nodes.^3^ The open left thoracoabdominal esophagectomy (LTE) includes a left-sided thoracotomy, which is extended into the abdomen. The diaphragm is opened which creates excellent exposure of the diaphragmatic hiatus and gastroesophageal junction.^4^^,^^5^ The approach is selectively applied at some high-volume esophageal surgery centers, but not all surgeons are familiar with the LTE although it has been demonstrated to be associated with important benefits and favorable postoperative course.^6^ The aim of the current study was to investigate postoperative results of LTE patients in a large contemporary international dataset and to assess whether the LTE remains a good option in selected patients undergoing surgical resection for esophageal cancer.

## METHODS

An international cohort study based on prospectively collected databases from six high-volume expert centers for esophageal cancer treatment. All patients treated with LTE from 2012 to 2022 were included in the study cohort. Detailed information about patient, tumor characteristics, and treatment were centralized in a single database. Data sharing was done in compliance with institutional IRBs and patient consent was waived as the study was retrospective and the individual patient information was anonymized. Participation in the study was reviewed and approved by the Institutional Review Board in all participating centers.

### Surgical technique

**Fig. 1:**
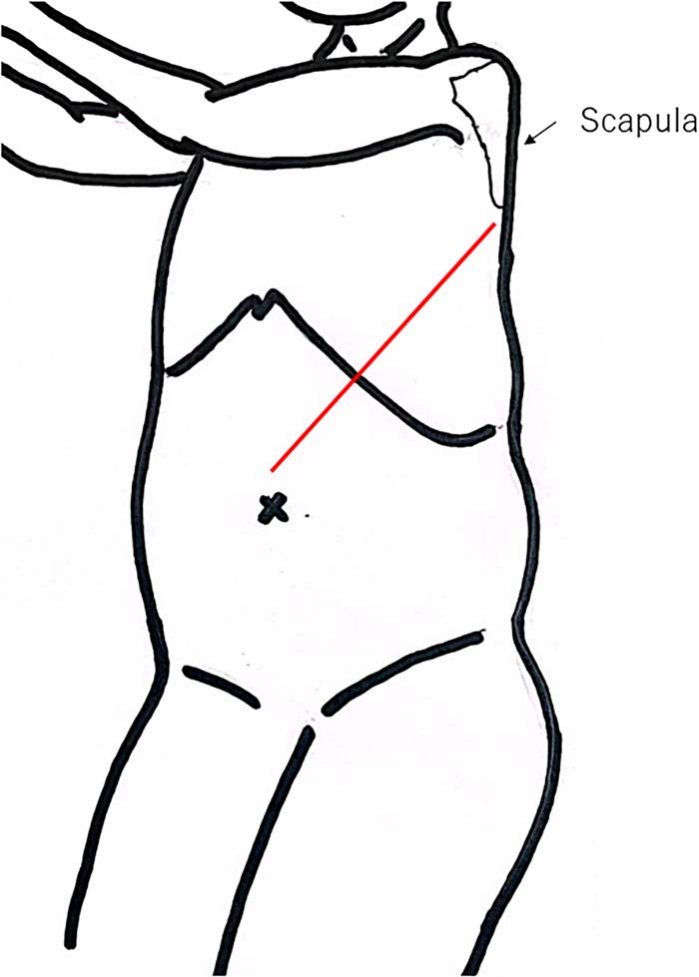
Schematic figure of patient position and incision for the LTE.

The LTE is performed with the patient positioned on their right side with both arms facing forward. The chest is tilted back ~30° to open up the left chest for improved access. The incision is placed as a left sided thoracotomy under the scapula in the 5th or 6th intercostal space. The incision starts at the base of the rib and ends at the midline of the upper abdomen just above the umbilicus. The chest and abdomen are opened, and the diaphragm is incised sparing the phrenic nerve. See Figure 1 and Supplemental Figure 1–6. Anastomoses were in the majority of patients done behind or above the aortic arch or in the neck; all of which would be above the azygous vein.

Indications for the LTE included gastro-esophageal junction tumors (Siewert type II or III) where an extended gastrectomy or Ivor Lewis esophagectomy was assessed to be less suitable or in other patients when assessed by the surgeon that the LTE would give the best opportunity for successful outcomes, for example large bulky tumors or tumors infiltrating both the stomach and the esophagus making it difficult to decide technique for reconstruction beforehand.

### Co-variates

Patient characteristics regarding, tumor, and treatment details were recorded prospectively in each participating center’s institutional database and compiled in a de-identified data base. Variables included age, gender, American Society for Anesthesiologists (ASA) score, Charlson Comorbidity Index, body weight, ECOG performance status, tumor location, clinical and pathological tumor stage, neoadjuvant treatment, and anastomosis level.

### Outcomes

Postoperative complications included: anastomotic leak, gastric conduit necrosis, ileus, small bowel obstruction, feeding J-tube complication, postoperative bleeding, chylothorax, recurrent laryngeal nerve paralysis, respiratory failure, pneumonia, pleural effusion, chest drain for air leak >10 days, septicemia, pulmonary embolism, deep vein thrombosis, renal failure, atrial fibrillation, and stroke. The ECCG complication definitions and the Clavien–Dindo scoring system were applied.^7^^,^^8^ Re-operations and length of hospital stay were recorded. Oncological results documented tumor-free (R0) resection margin according to the American definition, and number of resected lymph nodes, as well as ratio of positive versus negative lymph nodes.

### Statistical methods

Continuous outcomes were reported as median and interquartile range. Categorical values were reported as counts and percentages. A cox proportional hazard model was performed to analyze factors associated with survival. Univariable and multivariable adjusted results are presented as hazard ratios with 95% confidence intervals (CI). Gender, ASA-score, clinical tumor stage, and clinical lymph node stage were included in the model. Significance level was set at 0.05. Analyses were performed using STATA® version 13 software (StataCorp LP, College Station, Texas, USA).

## RESULTS

The study included 793 patients treated with LTE during the study period at the six participating centers. The largest number of operations from one center was 274 (34.6%), followed by 166 (20.9%) patients, 138 (17.4%) patients, 90 (11.4) patients, 80 (10.1) patients, and 45 (5.7%) patients from the smallest center. Median age was 65 years and 81.7% of the study participants were male, 90.5% of the patients had adenocarcinoma, 6.6% had squamous cell carcinoma, and 2.9% had other tumor types. The operation was performed in patients with a body weight ranging from 39 to 161 kg, the median weight was 80 kg (Table 1). The majority of patients had stage three esophageal cancer located in the distal esophagus or gastro-esophageal junction. More than 85% of the patients were treated with neoadjuvant therapy. Tumor-free resection margins were achieved in 89.8% of patients, median total number of harvested lymph nodes was 25 (Table 2).

**Table 1:** Baseline characteristics of patients undergoing LTE

***n (%)***		
Total		793 (100)
Age, median (range)		65 (26–91)
Male		645 (81.7)
Female		148 (18.3)
Body weight, median kg (range)		80 (39–161)
Preoperative comorbidity		
	No	310 (39.1)
	Yes	483 (60.9)
ECOG performance status		
	0	388 (60.1)
	1	217 (33.6)
	2	38 (5.9)
	3	3 (0.5)
	Missing	142
ASA score		
	I	42 (5.8)
	II	416 (57.1)
	III	267 (36.7)
	IV	3 (0.4)
	Missing	65
	Proximal esophagus	0 (0.0)
	Middle esophagus	43 (5.4)
	Distal esophagus	359 (45.2)
	GE junction Siewert type II	299 (37.7)
	GE junction Siewert type III	92 (11.6)
	Missing	0
Tumor histology		
	Adenocarcinoma	718 (90.5)
	Squamous cell carcinoma	52 (6.6)
	Other	23 (2.9)
Clinical tumor stage		
	T1	22 (2.8)
	T2	132 (16.8)
	T3	553 (70.5)
	T4a	78 (9.9)
	Tx	8
Clinical nodal stage		
	N0	265 (33.8)
	N1	355 (45.3)
	N2	129 (16.5)
	N3	34 (4.3)
	Nx	10

**Table 2:** Tumor characteristics and treatment details of patients undergoing LTE for esophageal cancer

***n (%)***		
Preoperative treatment		
	Surgery alone	104 (13.4)
	Neoadjuvant chemotherapy	513 (65.9)
	Neoadjuvant chemoradiotherapy	162 (20.8)
	Missing	14
Anastomosis level		
	Thoracic	719 (90.8)
	Cervical	73 (9.2)
	Missing	1
Tumor free resection margin†		
	Yes (R0)	707 (89.8)
	No (R1)	77 (9.8)
	No (R2)	3 (0.4)
	Missing	6
Pathologic tumor stage		
	(y)pT0 (complete histologic response)	74 (9.5)
	(y)pT1	94 (12.0)
	(y)pT2	120 (15.3)
	(y)pT3	422 (53.9)
	(y)pT4a	73 (9.3)
	(y)pTx	10
Pathologic nodal stage		
	(y)pN0	333 (42.6)
	(y)pN1	177 (22.6)
	(y)pN2	133 (17.0)
	(y)pN3	139 (17.8)
	(y)pNx	11
Number of resected lymph nodes, median (IQR)		25 (18–35)
Number of metastatic lymph nodes, median (IQR)		1 (0–4)

^†^According to the American definition for R0 resection*.*

In total 340/727 (46.8%) patients had a postoperative complication. The most common complication was pneumonia in 185/727 (25.5%) patients and atrial fibrillation in 91/727 (12.5%). Anastomotic leak occurred in 35/727 (4.8%) patients; no patient suffered from conduit necrosis. Clavien–Dindo score was available in 315/340 (92.6%) patients with postoperative complications. Clavien–Dindo score IIIa or higher occurred in 122/702 (17.4%) patients, and Clavien–Dindo score IIIb or higher in 75/702 (10.7%) patients. Thirty-day mortality occurred in 15/785 (1.9%) patients, and 90-day mortality in 39/785 (5.0%) patients (Table 3).

**Table 3:** Postoperative complications after LTE for esophageal cancer

***n (%)***		
Postoperative complication		340/727 (46.8)
	Missing data	66
	Anastomotic leak	35/727 (4.8)
	Gastric conduit necrosis	0/727 (0.0)
	Ileus	12/727 (1.7)
	Small bowel obstruction	4/727 (0.6)
	Feeding J-tube complication	7/727 (1.0)
	Postoperative bleeding	1/727 (0.1)
	Chylothorax	41/727 (5.6)
	Recurrent laryngeal nerve paralysis	1/727 (0.1)
	Respiratory failure	36/727 (4.9)
	Pneumonia	185/727 (25.5)
	Pleural effusion	58/727 (8.0)
	Chest drain for air leak >10 days	0/727 (0.0)
	Septicemia	14/727 (1.9)
	Pulmonary embolism	10/727 (1.4)
	Deep vein thrombosis	8/727 (1.1)
	Renal failure	0/727 (0.0)
	Atrial fibrillation	91/727 (12.5)
	Stroke	1/727 (0.1)
Clavien–Dindo score		
	I	18/703 (2.6)
	II	185/703 (26.3)
	IIIa	47/703 (6.7)
	IIIb	18/703 (2.5)
	IVa	43/703 (6.1)
	IVb	4/703 (0.1)
	V	10/703 (1.4)
	Missing	26
Clavien–Dindo score ≥ IIIa		122/702 (17.4)
Clavien–Dindo score ≥ IIIb		75/702 (10.7)
Surgical re-intervention for any cause		50/793 (6.3)
ICU re-admission		41/623 (6.6)
Median length of hospital stay in days (IQR)		10 (7–14)
Missing data hospital stay		29
30-day mortality		15/785 (1.9)
90-day mortality		39/785 (5.0)
1-year survival		564/697 (80.9)
3-year survival		258/556 (46.4)
5-year survival		132/487 (27.1)

**Fig. 2:**
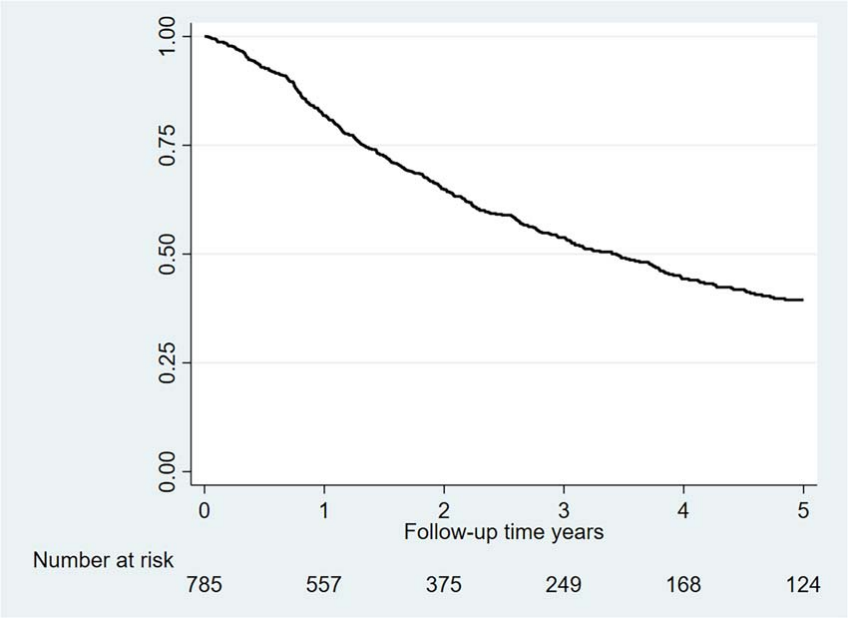
Survival after LTE for cancer.

**Fig. 3:**
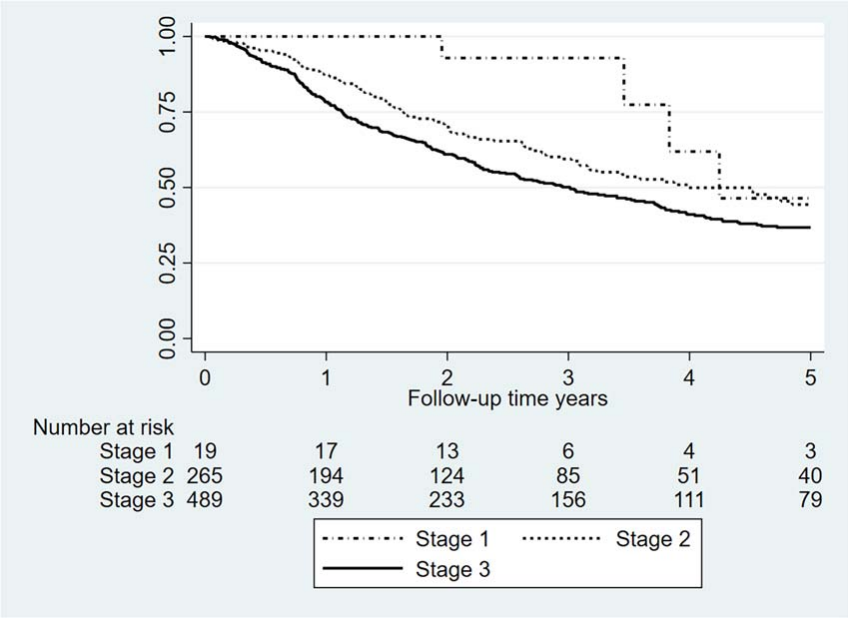
Survival after LTE for cancer stratified by tumor stage.

Factors with statistically significant association with survival in a univariable model were ASA-score, tumor location, tumor stage, tumor free resection margins, and anastomotic leak Neoadjuvant therapy was not associated with increased survival compared to surgery alone in both the univariable and multivariable adjusted model. However, neoadjuvant chemoradiotherapy compared to neoadjuvant chemotherapy showed statistically significant improved survival with adjusted hazard ratio: 0.60 (95% CI: 0.44–0.80, *P* = 0.001) in Table 4. Overall long-term survival is demonstrated in Figure 2, Figure 3 shows survival stratified by tumor stage.

**Table 4:** Cox proportional regression model of factors associated with overall survival after LTE for cancer

*Hazard ratio* *(95%* CI*)*	*Unadjusted*	*P-value*	*Multivariable adjusted* †	*P-value*
Male gender compared to female	1.27 (0.96–1.69)	0.092	1.21 (0.90–1.63)	0.214
Comorbidity	1.12 (0.90–1.39)	0.194	1.12 (0.89–1.42)	0.333
ECOG performance status	1.13 (0.94–1.36)	0.189	1.10 (0.91–1.35)	0.324
ASA score	1.26 (1.03–1.54)	0.025	1.26 (1.03–1.54)	0.027
Junction tumor compared to esophageal tumor	1.35 (1.09–1.67)	0.005	1.23 (0.98–1.54)	0.074
Clinical tumor stage (risk per increased stage)	1.37 (1.13–1.65)	0.001	1.32 (1.07–1.62)	0.009
Clinical N+ compared to N-	1.45 (1.14–1.85)	0.003	1.22 (0.97–1.30)	0.115
Neoadjuvant therapy compared to surgery alone	0.98 (0.72–1.34)	0.902	0.73 (0.50–1.07)	0.107
Neoadjuvant chemoradiotherapy versus chemotherapy	0.66 (0.50–0.88)	0.004	0.60 (0.44–0.80)	0.001
Tumor involved resection margin	2.55 (1.94–3.34)	<0.001	2.59 (1.91–3.51)	<0.001
Postoperative complication	1.03 (0.83–1.29)	0.791	1.00 (0.79–1.27)	0.996
Anastomotic leak	1.64 (1.02–2.65)	0.041	1.62 (0.99–2.66)	0.054
Chylothorax	0.92 (0.55–1.55)	0.767	0.98 (0.58–1.65)	0.940
Pneumonia	1.15 (0.90–1.46)	0.279	1.24 (0.96–1.61)	0.097
Operating center	0.98 (0.91–1.05)	0.560	0.96 (0.89–1.04)	0.318

^†^Adjusted for gender, ASA-score, clinical tumor stage, and clinical lymph node stage

## DISCUSSION

This study has compiled the largest cohort of LTE patients published to date and the results demonstrate that LTE is a safe and effective approach for esophageal and gastro-esophageal junction cancer treatment in selected patients when performed at high-volume expert centers. The level of postoperative complications compares well to what is reported in the literature after other types of minimally invasive and open procedures for esophageal and gastro-esophageal junction cancer.^3^^,^^6^ With respect to anastomotic leak rate and overall complication rate the results of the current study are significantly better than results published in contemporary large international datasets.^3^ The fact that no patients suffered from gastric conduit necrosis and that less than 5% had anastomotic leak shows that the technique is associated with a favorable postoperative recovery. There is a perception that the thoraco-abdominal incision that the LTE requires is associated with increased postoperative morbidity. A Japanese randomized controlled trial compared left thoracoabdominal to transhiatal approach for total gastrectomy for gastroesophageal junction cancers. The study showed increased risk for postoperative morbidity and decreased survival in the left thoracoabdominal group and concluded that the approach should not be used.^9^^,^^10^ However, the results of the current study demonstrate that the incision offers an opportunity for excellent tumor dissection which increases the chance for tumor free resection margins and safe anastomotic techniques with relatively low risk of postoperative complications considering that most of the included patients had locally advanced tumors.^11^

Neoadjuvant therapy was not associated with overall increased survival rate which has been shown in other large cohort studies, this is probably explained by patient selection and sample size of patients treated with surgery alone.^12^ Neoadjuvant chemoradiotherapy was associated with improved survival compared to neoadjuvant chemotherapy even after adjusting for tumor stage. This might be explained by the fact that many patients had large locally advanced tumors and that neoadjuvant chemoradiotherapy increases the chance for tumor free resection margins compared to neoadjuvant chemotherapy and surgery alone.^12–14^ However, the study was not designed to evaluate neoadjuvant therapy and these findings should be interpreted with caution.

Historically, limitation of the LTE procedure includes an atypical incision which requires a specific learning curve, and that the incision may be associated with increased postoperative pain especially when compared to current minimally invasive techniques. Advantages of the LTE include the fact that the incision while extensive, typically occupies only a single dermatome which makes it highly amenable to postoperative pain management with epidural or paravertebral pain catheters. In addition, the LTE provides optimal exposure for large, complicated tumors with potential invasion of the posterior pericardium, spine and descending thoracic aorta, although the technique is not applicable for tumors extending behind and proximal to the aortic arch. The LTE provides optimal exposure within the upper abdomen for patients with profound hepatomegaly. It is also the only surgical approach that provides simultaneous access to chest and abdominal cavities, allowing a unique opportunity for intraoperative assessment of resection margins and anastomotic location. As a result, the LTE is a very flexible approach that facilitates intraoperative reconstruction modification since it can be easily switched from construction of a gastric conduit to either a Roux-en-Y jejunal or colonic reconstruction. In addition, anastomotic location can vary between placing it behind the aortic arch, just below the thoracic inlet above the aortic arch or several centimeters higher above the thoracic inlet with the addition of a separate cervical component to the operation. The procedure might be associated with decreased incidence of gastric dilation into the right chest as the gastric conduit is typically located well within the mediastinum and not within the right thoracic cavity as occurs in Ivor Lewis operations.

The study has some limitations that should be recognized, for example it does not include a comparison group, which is explained by the fact that it is not possible to find a suitable matched patient cohort for comparison with the current study design. Patients selected for the LTE most commonly have complex tumors in the gastroesophageal junction but the indication for the operation is likely to differ to some degree in the included centers. Details about postoperative pain management were not collected. Previous studies have compared LTE to two-stage transthoracic esophagectomy and minimally invasive esophagectomy with comparable results.^4^^,^^6^ A randomized controlled trial would be required to generate a valid group for comparison but in the current environment, a randomized trial of an open versus minimally invasive approach, even in a selected patient population, would be difficult, at best, and likely impossible to perform. Follow-up of patients´ health-related quality of life would improve the quality of the analyses but was unfortunately not available in this study.

In conclusion, this study demonstrates that LTE can be applied with favorable results in selected patients with advanced esophageal or gastro-esophageal cancer at high-volume centers. This should provide an incentive for gastro-esophageal surgeons to become acquainted with the LTE for the operation to be part of the operative treatment armamentarium in high-volume centers.

## Supplementary Material

supp1_doae039

supp2_doae039

supp3_doae039

supp4_doae039

supp5_doae039

supp6_doae039
