# Supplementary figures and images for: Contemporary outcomes of left thoraco-abdominal esophagectomy due to cancer in the esophagus or gastroesophageal junction, a multicenter cohort study
Source: Dis Esophagus. 2024 Apr 28;37(9):doae039. doi: 10.1093/dote/doae039 (PMC11360984; doi:10.1093/dote/doae039)

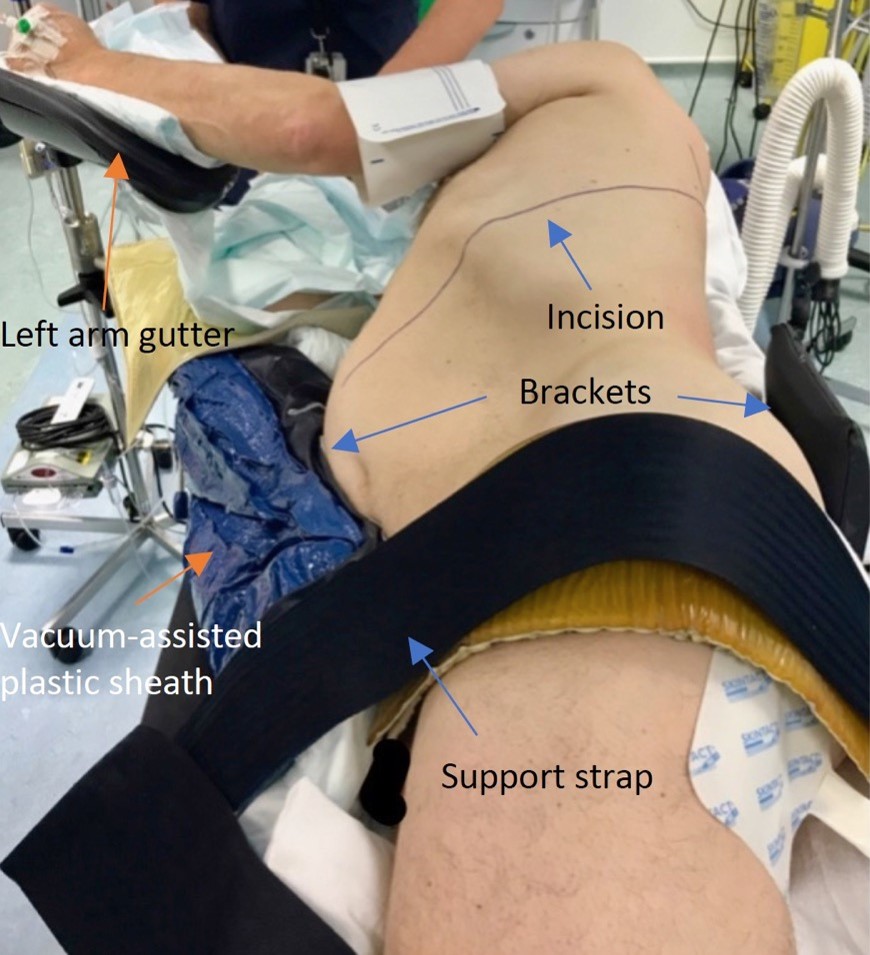

Supplement: supp1_doae039 [file supp1_doae039.jpeg]

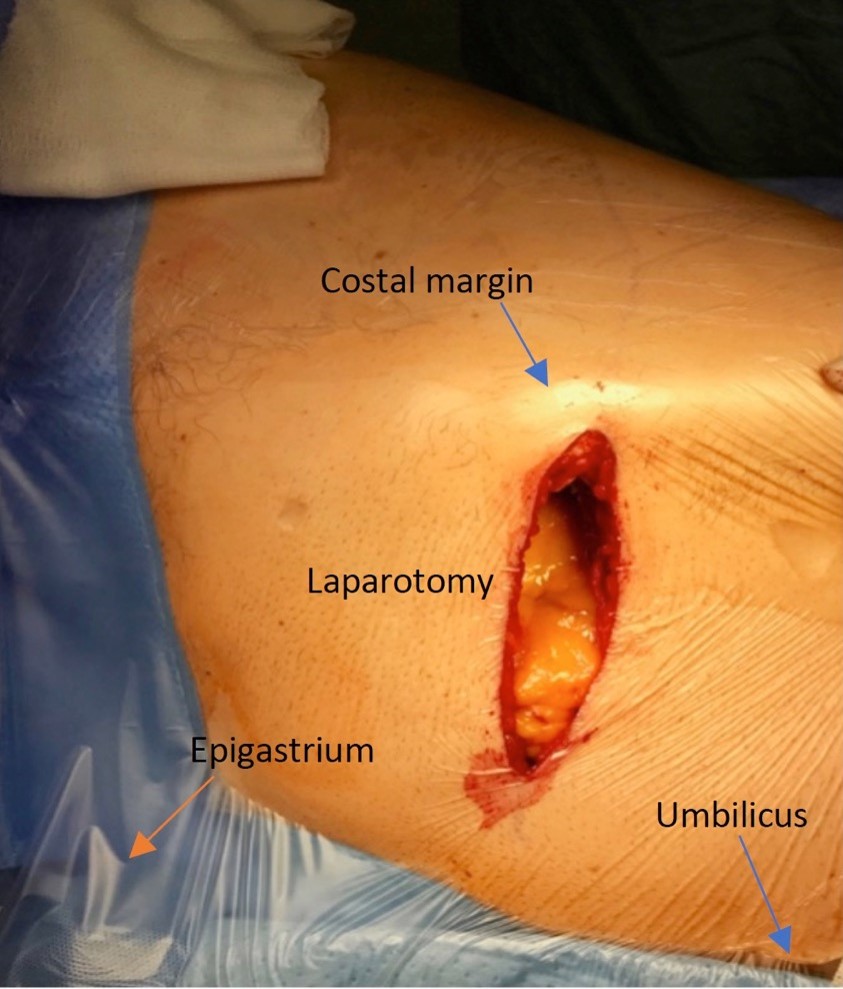

Supplement: supp2_doae039 [file supp2_doae039.jpeg]

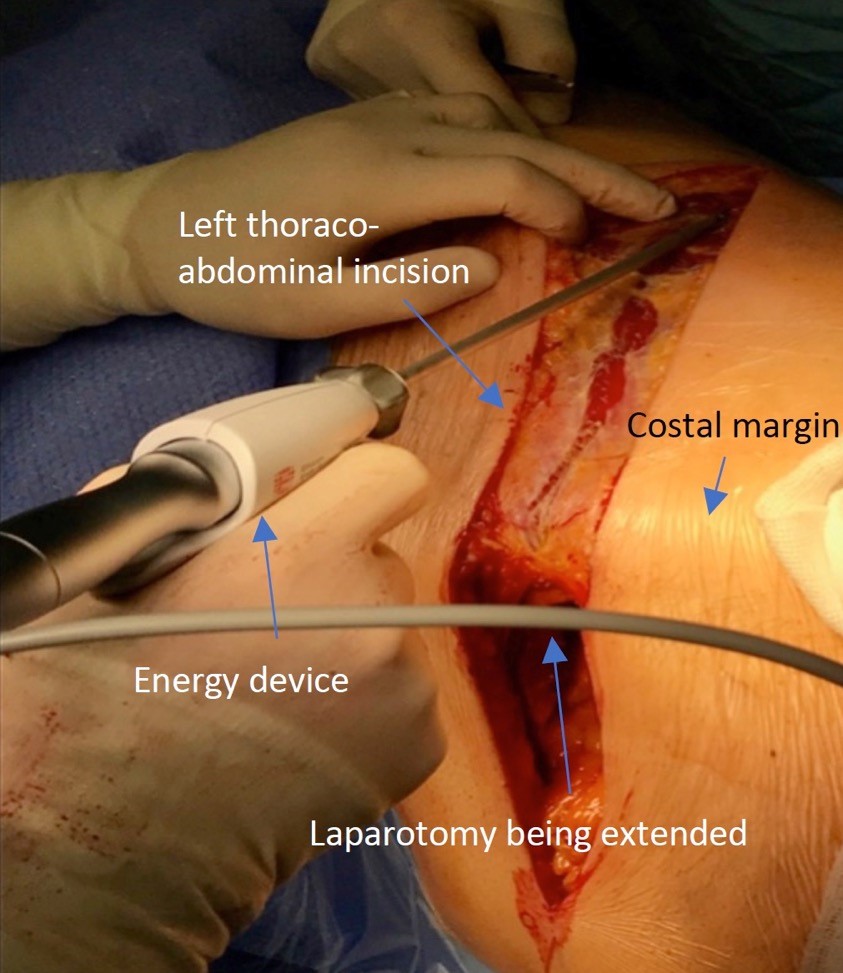

Supplement: supp3_doae039 [file supp3_doae039.jpeg]

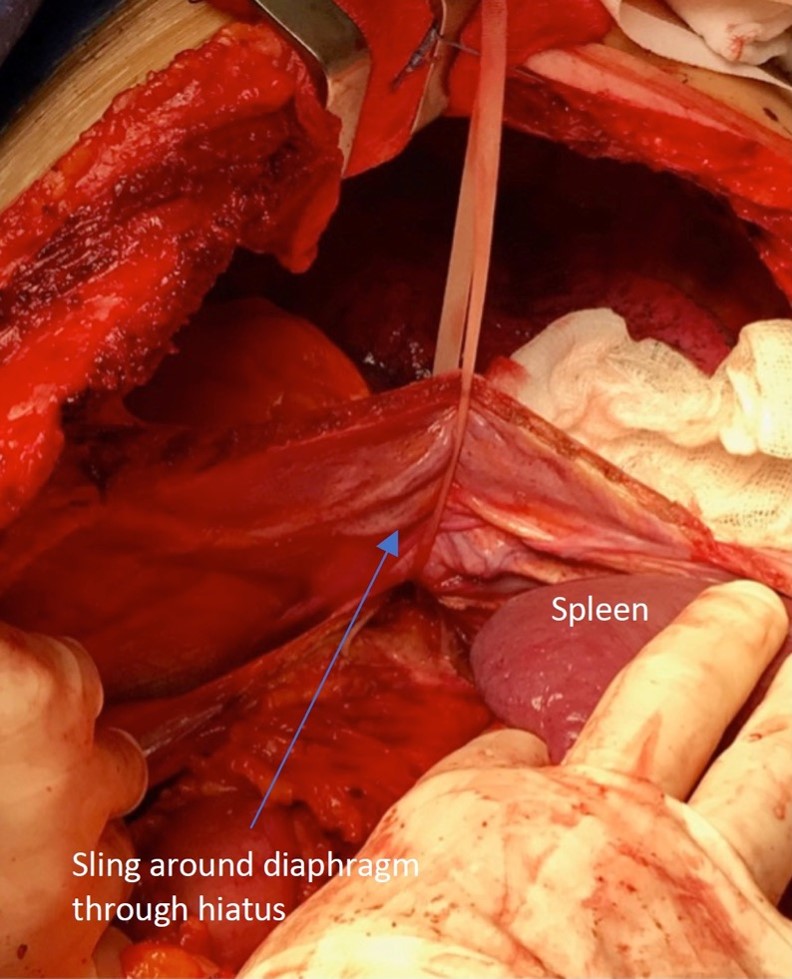

Supplement: supp4_doae039 [file supp4_doae039.jpeg]

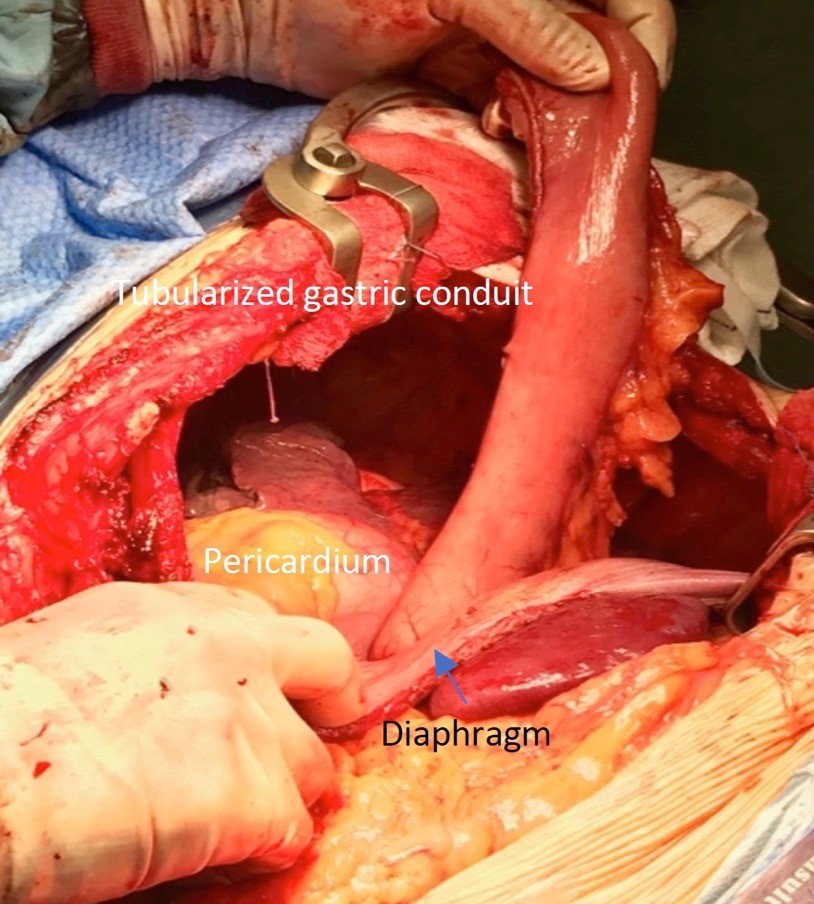

Supplement: supp5_doae039 [file supp5_doae039.jpeg]

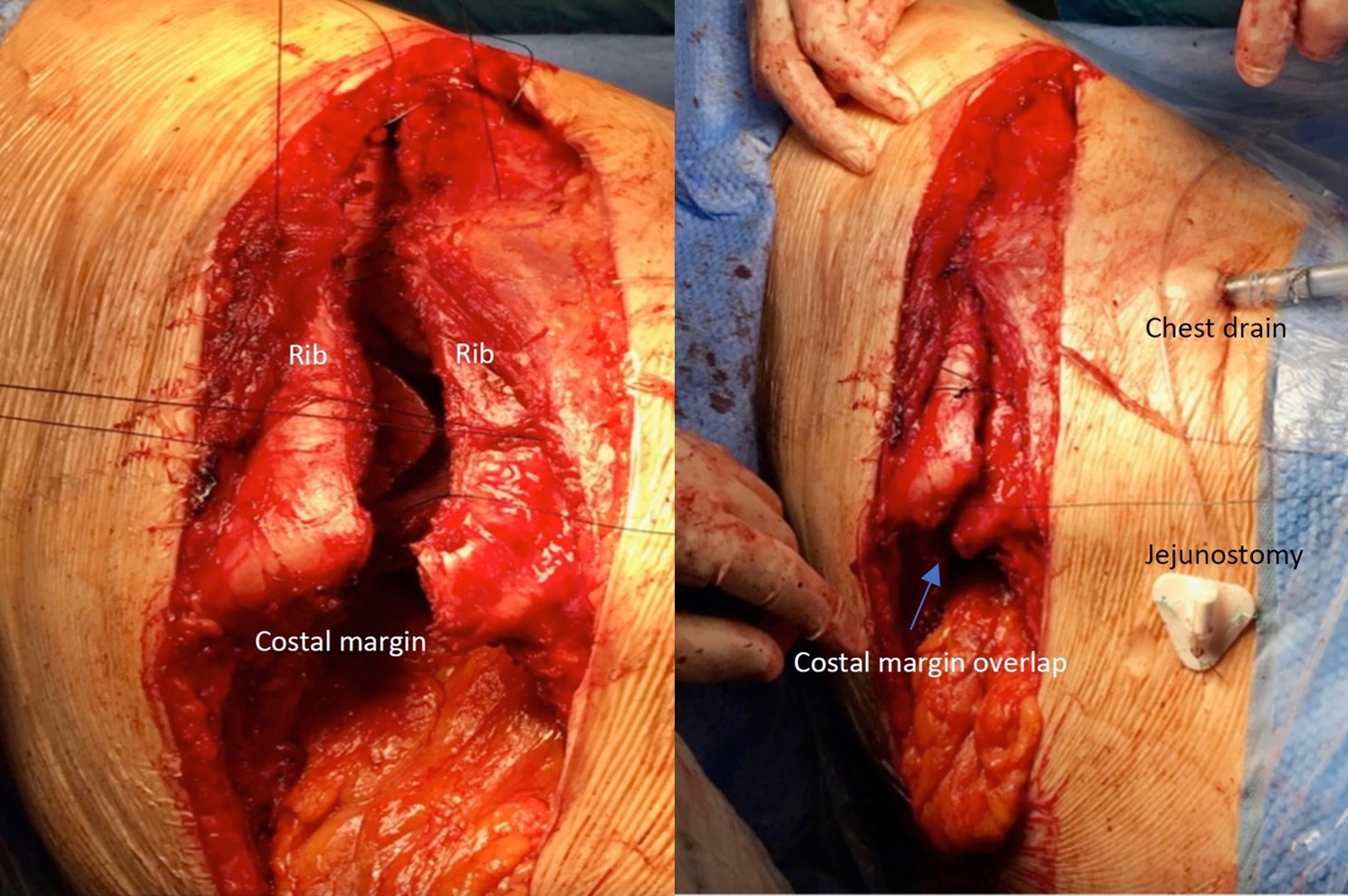

Supplement: supp6_doae039 [file supp6_doae039.jpeg]
